# Measuring How Recombination Re-shapes the Evolutionary History of PRRSV-2: A Genome-Based Phylodynamic Analysis of the Emergence of a Novel PRRSV-2 Variant

**DOI:** 10.3389/fvets.2022.846904

**Published:** 2022-03-25

**Authors:** Nakarin Pamornchainavakul, Mariana Kikuti, Igor A. D. Paploski, Dennis N. Makau, Albert Rovira, Cesar A. Corzo, Kimberly VanderWaal

**Affiliations:** Department of Veterinary Population Medicine, University of Minnesota, Saint Paul, MN, United States

**Keywords:** porcine reproductive and respiratory syndrome virus 2, viral recombination, whole genome sequencing, variant emergence, epidemics

## Abstract

While the widespread and endemic circulation of porcine reproductive and respiratory syndrome virus type 2 (PRRSV-2) causes persistent economic losses to the U.S. swine industry, unusual increases of severe cases associated with the emergence of new genetic variants are a major source of concern for pork producers. Between 2020 and 2021, such an event occurred across pig production sites in the Midwestern U.S. The emerging viral clade is referred to as the novel sub-lineage 1C (L1C) 1-4-4 variant. This genetic classification is based on the open reading frame 5 (ORF5) gene. However, although whole genome sequence (WGS) suggested that this variant represented the emergence of a new strain, the true evolutionary history of this variant remains unclear. To better elucidate the variant's evolutionary history, we conducted a recombination detection analysis, time-scaled phylogenetic estimation, and discrete trait analysis on a set of L1C-1-4-4 WGSs (*n* = 19) alongside other publicly published WGSs (*n* = 232) collected over a 26-year period (1995–2021). Results from various methodologies consistently suggest that the novel L1C variant was a descendant of a recombinant ancestor characterized by recombination at the ORF1a gene between two segments that would be otherwise classified as L1C and L1A in the ORF5 gene. Based on analysis of different WGS fragments, the L1C-1-4-4 variant descended from an ancestor that existed around late 2018 to early 2019, with relatively high substitution rates in the proximal ORF1a as well as ORF5 regions. Two viruses from 2018 were found to be the closest relatives to the 2020-21 outbreak strain but had different recombination profiles, suggesting that these viruses were not direct ancestors. We also assessed the overall frequency of putative recombination amongst ORF5 and other parts of the genome and found that recombination events which leave detectable numbers of descendants are not common. However, the rapid spread and high virulence of the L1C-1-4-4 recombinant variant demonstrates that inter-sub-lineage recombination occasionally found amongst the U.S. PRRSV-2 might be an evolutionary mechanisms that contributed to this emergence. More generally, recombination amongst PRRSV-2 accelerates genetic change and increases the chance of the emergence of high fitness variants.

## Introduction

Over the three decades since its initial report, porcine reproductive and respiratory syndrome (PRRS) has undermined the stability of U.S. swine production both in terms of herd health and economics (1). The disease devastates pig production by causing reproductive failure in breeding herds and respiratory associated morbidity/mortality in growing pigs leading to poor production performance (2). In the U.S., outbreaks are mostly classified as porcine reproductive and respiratory syndrome virus 2 (PRRSV-2). The causative agent of PRRSV-2 is *Betaarterivirus suid 2*, an enveloped RNA virus belonging to *Arteriviridae* family in the *Nidovirales* order whose virulence varies by strain (3, 4). Due to lack of RNA proofreading during replication, PRRSV-2 has a high mutation rate even amongst RNA viruses (5, 6). Additionally, recombination–a process where genomic parts are exchanged between viral variants co-infecting a cell–can occasionally contribute to PRRSV-2 genetic variation. RNA viruses including PRRSV-2, are prone to recombination processes such as template switching in sub-genomic RNA synthesis (7–9).

Recently, pig producers in the Midwestern U.S. experienced atypical production losses caused by a fast-spreading variant of PRRSV-2 (10). From early 2020 to September 2021, 355 genetically similar viruses were detected (i.e., > 98% nucleotide identity based on the open reading frame 5–ORF5–gene). Based on data from the Morrison Swine Health Monitoring Project (MSHMP), which tracks the infection status of ~50% of the U.S. breeding herd (cite Perez–Voluntary data sharing), 294 pig sites belonging to 15 different production systems, and ~12% of breeding farms in the region had been impacted (11). The virus involved in this outbreak is referred to as a novel L1C-1-4-4 variant, as it falls within the L1C sub-lineage based on phylogenetic relatedness (12) and mostly possesses a 1-4-4 cut-pattern based on conventional restricted fragment length polymorphism (RFLP)-based classification. Both classifications are based on ORF5 sequences (13). While the exact case definition used is based on >98% nucleotide identity on ORF5 (10), here we refer to this variant simply as L1C-1-4-4. Although ORF5 has been widely used for virus classification or epidemiological assessment since it is highly variable and immunologically relevant (14), it only represents ~4% of the genome and may not represent the full evolutionary history of the virus, particularly when recombination is involved. For example, when phylogenetic trees are constructed using whole genome sequences, the L1C-1-4-4 variant nests within a clade of viruses that are classified as sub-lineage L1A based on the ORF5 gene (10, 15), suggesting that recombination may be confounding the virus' genealogical tree topology. Because there is no WGS-based nomenclature for classifying PRRSV-2 genetic sub-types, here we refer to WGSs according to their ORF5 lineage classification and recognizing the limitation that genetic relatedness might not hold true when looking at other regions of the genome.

The rapid spread and high production impact of the newly emerged L1C-1-4-4 variant has drawn concern from the industry as similar events occurred in the past and the contributing factors to emergence of these strains are poorly understood. Given that PRRSV-2 in the U.S. is characterized by the cyclic emergence of new strains, and turnover in the dominant sub-lineage every few years (16), this emerging variant may continue increasing in prevalence, bringing further issues to the U.S. swine industry. However, previous emergence events have largely been documented based on ORF5 sequence data, which limits our ability to fully discern evolutionary processes associated with strain emergence. For example, PRRSV-2 virulence and antigenic determinants are multigenic, meaning that clinical presentation characteristics are influenced by a variety of genes throughout the viral genome (4). Thus, understanding the origin of this variant from a whole genome perspective is a crucial step in response to this ongoing outbreak, and may help elucidate evolutionary processes associated with strain emergence more broadly and lead to potential preventive interventions at the farm level. Here, we estimate divergence times, mutation rates, and parental strains of the novel L1C-1-4-4 variant using genomics-based approaches. More generally, to better understand the role of recombination in shaping PRRSV-2 phylogenies, we also quantify the frequency of potential inter- and intra-lineage recombination events.

## Methods

### Data

A convenience sample of PRRSV-2 L1C-1-4-4 variant whole genome sequences (WGS) were obtained from the Veterinary Diagnostic Laboratory at the University of Minnesota [see (10) for details]. These samples were from multiple production systems in the Midwestern U.S. participating in the Morrison Swine Health Monitoring Project. Sample selection criteria for WGS included having ORF5 sequences within the emerging variant's phylogenetic clade (<2% genetic distance to at least one other sequence classified as L1C-1-4-4) and cycle threshold (Ct) value ≤25 for reverse transcription polymerase chain reaction (RT-PCR) using VetMAX™ NA and EU PRRSV Reagents (Thermo Fisher Scientific, MA, USA). Oral fluids and processing fluids samples were excluded due to the low success rate for whole genome sequencing (17, 18). At least one ORF5 sequence from each participating system was whole-genome sequenced. For systems that had two or more ORF5 L1C-1-4-4 sequences identified during this period, the earliest and the most recent samples were selected for WGS sequencing. As described in Kikuti et al. (10), the selected samples were sequenced using Clontech SMARTer RNA Pico v2 kit on illumina MiSeq v3 (Illumina, CA, USA). Of the total 19 WGSs (GenBank accession numbers OL963961-OL963979), one isolate classified as the novel L1C-1-4-4 variant based on the above criteria was from an outbreak limited to a single production system in 2018, while the others were collected from multiple systems during the current epidemic (i.e., 2020–2021). We aligned the WGSs with all available PRRSV-2 WGSs from the U.S. that were publicly available and included date meta-data from NCBI GenBank (*n* = 232, Supplementary Table 1), ranging between 1995 and 2018 using MAFFT (19) and manual curation. Genetic distances were calculated between all sequences using seqcombo (20).

### Recombination Detection

As a first step for screening sequences for recombination, the alignment was imported to RDP5 (21) for recombination detection. A putative recombination event was flagged when it was detected by at least four of seven methods: RDP (22), GENECONV (23), MaxChi (24), BootScan (25), SiScan (26), Chimaera (27), and 3Seq (28). We performed the analysis as a two-pronged approach. First, we specifically explored recombination in the novel L1C-1-4-4 variant group, which was set as a query against all GenBank WGS references. Second, the alignment was fully scanned (with no reference and query groups defined) to estimate the location of recombination hotspots within the genome. A recombination hotspot is defined as a genomic position in which the frequency of putative recombination exceeds neutral expectations (>99% confidence interval of the local density plot created by a permutation test) (29); genomic regions between hotspots are inferred to have low rates of recombination. Thus, the locations of hotspots can be used to subdivide the genome into fragments, where each fragment is relatively free of frequent within-fragment recombination and thus can be used for further phylogenetic analysis (30, 31).

Maximum likelihood phylogenies were built from each WGS fragment using W-IQ-TREE (32) with automated substitution model selection and 1,000 bootstraps. The consensus trees were assessed to: (1) check the temporal signal under a molecular clock assumption using TempEst (33), and any fragment whose phylogenetic reconstruction did not show a sufficient temporal signal was excluded from further time-scaled analyses. (2) Down-sample the dataset based on pairwise distances from the novel L1C-1-4-4 variant using *ape* version 5.5 (34) applied in R (35). Only the 50 most closely related sequences to each distinct fragment of the novel L1C-1-4-4 variant were retained, yielding a total of 142 sequences for further analysis (Supplementary Table 1).

### Time-Scaled Phylogenetic Reconstruction

The time to the most recent common ancestor (tMRCA) and substitution rate of each fragment were estimated by Bayesian inference with Markov chain Monte Carlo (MCMC) applied in BEAST v.1.10.4 (36). According to IQ-TREE's substitution model test, we chose the general time reversible (GTR) with empirical base frequencies and gamma plus invariant site (G + I) heterogeneity model for all fragments. An uncorrelated relaxed clock (37) with log-normal distribution and the Gaussian Markov random field (GMRF) skyride (38) were specified as molecular clock model and coalescent prior, respectively. The alignments with these model settings were run with 500 million generations of MCMC. Maximum clade credibility (MCC) trees of each fragment were built using TreeAnnotator v.1.10.4 and visualized on the Nextstrain platform (39).

### Discrete Trait Analysis

The frequency of inter- and intra-lineage recombination between ORF5 and other fragments was approximated through WGS-fragment phylogenies using discrete trait analysis in BEAST. For each WGS fragment, the ORF5-based lineage (14) or sub-lineage (12) of each sample was assigned as a discrete trait, and the ancestral trait of each internal node was inferred. Ancestral transitions between traits (i.e., the label the sequence received based on its ORF5 lineage) in the WGS-fragment phylogenies can be interpreted as putative recombination between the ORF5 gene and other WGS regions (i.e., instances where sequences are no longer clustered with other sequences that share the same ORF5-lineage label). Potential recombination in the WGS-fragment phylogenies were estimated from the number of trait (lineage) transitions with Bayes factors (BF) support obtained from an asymmetric substitution model with Bayesian stochastic search variable selection (BSSVS). Other parameters were set as the software default. The analyses were run with MCMC length of 500 million each. Ancestral states annotated on MCC trees were visualized using FigTree v.1.4.4 (40). Lineage and sub-lineage transitions were reported with BF computed by SpreaD3 (41). High numbers of transitions between inferred ORF5-lineage in the phylogenies of other WGS-fragments would provide support that recombination is more common, and that shared phylogenetic ancestry based on ORF5 lineage identity is scrambled on the whole genome due to putative recombination. Low transitions suggest that recombination events that leave descendants detected by surveillance activities are relatively rare, and that shared ancestry based on ORF5 lineages are relatively stable across the genome.

## Results

The 18 novel L1C-1-4-4 WGSs associated with the 2020-21 outbreak displayed a 98.2 to 99.9% nucleotide identity. The 2018 virus, which was included for whole genome sequencing based on its high similarity on ORF5, showed a 96.5 to 97.4 % pairwise similarity to the 2020-21 L1C-1-4-4 whole genomes. The greatest difference (<90 % similarity) between the 2020-21 group and the 2018 virus was in nsp9 to nsp10 in the ORF1b region (Supplementary Figure 1).

### Recombination Profile

**Figure 1:**
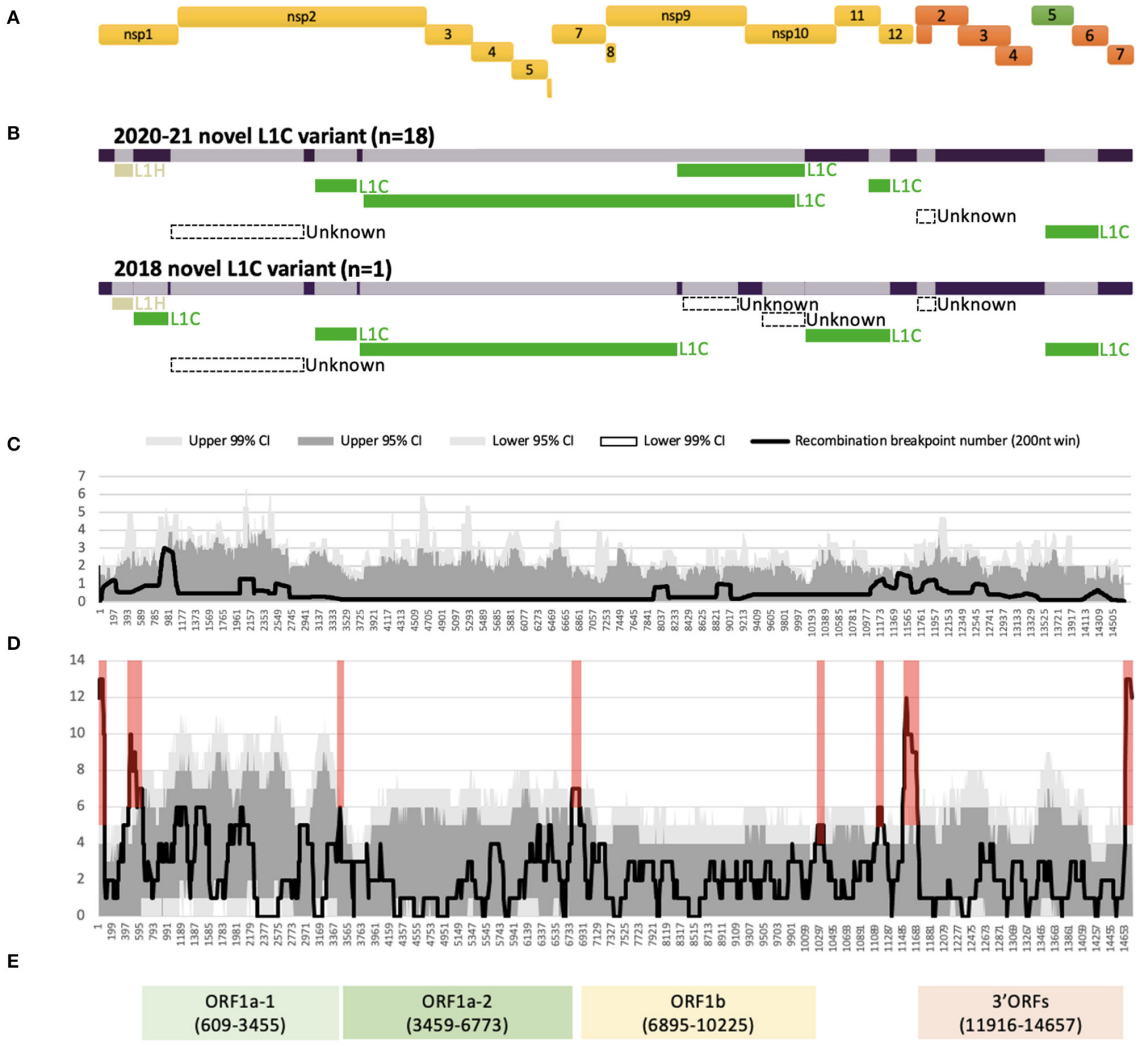
Recombination profile of the novel L1C-1-4-4 viruses in relation to PRRSV-2 genomic organization. **(A)** PRRSV-2 genomic organization. **(B)** Putative recombinant regions and minor parents of the 2020–21 (*n* = 18) and the 2018 (*n* = 1) L1C-1-4-4 variants. The long bar across the top represents the viral genomic backbone. The smaller bars below represent putative minor parents labeled according to the ORF5-based sub-lineages. **(C)** Recombination breakpoint distribution of the novel L1C-1-4-4 WGSs as queries against other PRRSV-2 WGSs. **(D)** Overall recombination breakpoint distribution of the 251 PRRSV-2 WGSs. Recombination hotspots defined by the local density plot are highlighted in red. **(E)** Genomic fragments with low within-fragment recombination rates used for phylogenetic analyses. Nucleotide positions in the alignment are shown in the parenthesis.

All 19 WGSs had a relatively similar recombination profile, with at least six putative recombinant regions in common across the viral genome. The minor parents (i.e., the parent contributing the shorter part of the overall sequence) of several of these recombinant regions were viruses that clustered with other viruses classified as sub-lineage L1C in their ORF5 gene. For all 19 WGSs, a large recombinant region was identified in nsp2. The recombination detection algorithms implemented using RDP5 were not able to identify a feasible minor parent in the alignment of the 232 GenBank sequences for the nsp2 recombinant region and for a short recombinant region in ORF2 genes. The detectable minor parents of other events were identified as viruses belonging to the L1H (in nsp1) and L1C (in nsps2-9, and ORFs5-6) sub-lineages based on their ORF5 gene variation (Figure 1B, Supplementary Table 2). Major differences in the recombination pattern of the 2020-21 (*n* = 18) and the 2018 (*n* = 1) samples were found in the nsp9 to nsp12 of ORF1b gene, where the parents of the 2020–2021 sequences were other L1C, while the 2018's parents were mostly unknown (Figure 1B, Supplementary Table 2). In agreement with the estimated location of recombinant genomic regions, recombination breakpoints of this variant are located in the following genomic regions: nsp1 flanking regions, insertion and deletion (indel) sites of nsp2 (42), inside nsp9, ORF1ab-ORF2 junction, and ORF2 and ORF5 flanking regions (Figures 1A,C).

Several recombination hotspots were detected when employing an all-to-all approach for identifying recombination events (Figure 1D). We used these hotspots to extract four fragments within which there was a low frequency of recombination, namely ORF1a-1 [nucleotide position in the alignment (nt) 609 to 3,455], ORF1a-2 (nt 3,459 to 6,773), ORF1b (nt 6,895 to 10,225), and 3' ORFs (nt 11,916 to 14,657) after their genomic annotation (Figure 1E). A significant temporal signal was found in the best-fitting rooted maximum likelihood trees for all fragments, though unlike the other fragments, the temporal signal of ORF1a-2 was only significant when using the correlation and R-squared-based rooting methods (Supplementary Table 3).

### Evolutionary Rate and Ancestral Date

Amongst time-scaled phylogenies of PRRSV-2 genomic fragments, mean evolutionary rates ranged from 2.40 to 3.81 × 10^−3^ substitutions/site/year, with the exception of ORF1a-2, which had the mean evolutionary rate 10 times lower than that of the rest of the genome (Table 1). ORF1a-2's temporal signal, as estimated by Tempest, was more uncertain which may be caused by the low evolutionary rate, ultimately resulting in an eccentric ancestral date estimation that may not be reliable. We thus excluded this fragment from the interpretation of time-scaled trees. The 161 viruses included in this analysis (L1C-1-4-4 variant and the most closely related GenBank sequences across each fragment) had median tMRCAs ranging from 1985 to 1989. The 2020–2021 novel L1C-1-4-4 samples (*n* = 18) form a monophyletic clade sharing a common ancestor in all WGS fragments' trees. Their tMRCA was dated from late 2018 to early 2019. If the 2018 sequence whose ORF5 gene had high nucleotide identity to the 2020–21 L1C-1-4-4 samples was included, tMRCA of the complete set of novel L1C-1-4-4 variants (*n* = 19) was estimated to be in 2017. The 2018 sequence was a basal taxon to L1C-1-4-4 clade in most fragments except the ORF1b tree, where the 2018 taxon was separated and embedded in a clade consisting of viruses labeled as the L1A sub-lineage, suggesting that the 2018 virus experienced a separate recombination event that did not occur in the 2020-21 sequences. The nucleotide substitution rate at the ancestral branch of the novel L1C (inclusive of the 2018 sequence) was lower in some fragments than the overall mean rate, whereas the ancestral branch of the more recent 2020–2021 epidemic samples had a higher rate than the mean (Table 1, trees in Nextstrain: “https://nextstrain.org/community/NakarinP/prrsv/”).

**Table 1:** Ancestral date and evolutionary rate estimates of the novel L1C-1-4-4 variants and other PRRSV-2.

**WGS fragment**	**Overall (*****n*** **= 161)**		**2020–2021 novel L1C-1-4-4 (*****n*** **= 18)**		**2018–2021 L1C-1-4-4 (*****n*** **= 19)**	
	tMRCA^*^ **(95% HPD)**	mean rate^**^ **(95% HPD)**	tMRCA^*^ **(95% HPD)**	ancestral branch rate^**^ **(95% HPD)**	tMRCA^*^ **(95% HPD)**	ancestral branch rate^**^ **(95% HPD)**
ORF1a-1	Oct 1988 (Mar 1983, April 1992)	3.81 × 10^−3^ (2.69 × 10^−3^, 4.98 × 10^−3^)	Nov 2018 (Feb 2018, Sep 2019)	2.15 × 10^−2^ (2.00 × 10^−3^, 6.79 × 10^−2^)	Nov 2017 (Jan 2016, Jun 2018)	1.22 × 10^−2^ (7.00 × 10^−4^, 4.60 × 10^−3^)
ORF1a-2^#^	Aug 1546^#^ (Jan 1194, Jan 1782)	4.07 × 10^−4^ (3.29 × 10^−4^, 4.86 × 10^−4^)	May 2003^#^ (Jun 1993, Mar 2014)	8.96 × 10^−4^ (1.22 × 10^−4^, 2.39 × 10^−3^)	Jun 1997^#^ (May 1987, Jan 2009)	2.92 × 10^−3^ (4.61 × 10^−4^, 6.78 × 10^−3^)
ORF1b	Oct 1985 (Feb 1979, Jun 1991)	2.40 × 10^−3^ (1.67 × 10^−3^, 3.07 × 10^−3^)	Jan 2019 (Apr 2018, Nov 2019)	8.82 × 10^−3^ (3.05 × 10^−3^, 1.52 × 10^−2^)	NA (the 2018 taxon does not group with others)	NA (the 2018 taxon does not group with others)
3'ORFs	Jul 1987 (Apr 1981, May 1992)	2.55 × 10^−3^ (1.91 × 10^−3^, 3.23 × 10^−3^)	Dec 2018 (Feb 2018, Sep 2019)	5.56 × 10^−3^ (6.64 × 10^−4^, 1.17 × 10^−2^)	May 2017 (July 2014, May 2018)	1.60 × 10^−3^ (7.94 × 10^−4^, 2.51 × 10^−3^)
ORF5	Nov 1989 (Oct 1984, May 1994)	3.20 × 10^−3^ (2.34 × 10^−3^, 4.09 × 10^−3^)	Dec 2018 (Mar 2018, Nov 2019)	5.15 × 10^−3^ (2.65 × 10^−4^, 1.27 × 10^−2^)	Sep 2017 (Aug 2015, Jun 2018)	2.04 × 10^−3^ (3.42 × 10^−4^, 3.99 × 10^−3^)

* *Time to the most recent common ancestor*.

** *Evolutionary rate (substitutions/nucleotide site/year)*.

# *Estimates may be anomalous due to relatively poor temporal signal in this fragment*.

### Inter- and Intra-lineage Recombination

**Figure 2:**
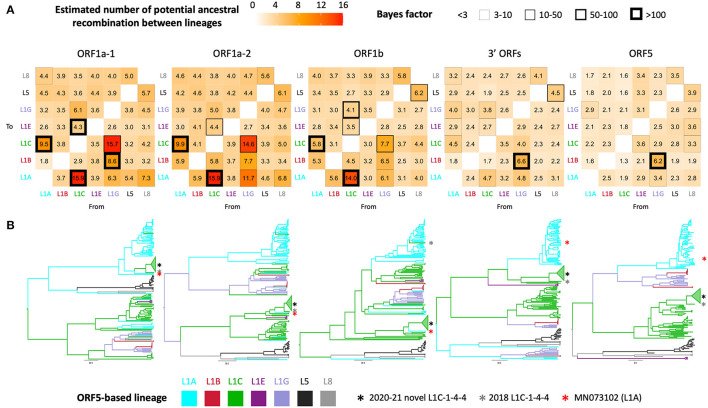
Discrete trait analysis of PRRSV-2 lineage/sub-lineage recombination. **(A)** Heat map showing number of potential ancestral recombination between lineages/sub-lineages of each genomic fragment estimated from the trait transitions. Cell border thickness represents Bayes factor (BF) support for each recombination. **(B)** Bayesian MCC trees colored by ancestral ORF5-based lineage or sub-lineage. Asterisks locate the phylogenetic position of taxa of interest.

Phylogenetic clustering of samples on each WGS-fragment tree, labeled according to ORF5 lineages, are visually well-aligned with ORF5-based lineage classification. However, there were instances where clustering of sequences by ORF5 lineage did not translate perfectly to other WGS-fragments, which suggested the possibility of genomic recombination outside ORF5 gene. In the ORF5 tree, there was no significant mixing between lineages/sub-lineages except between sub-lineage L1G ancestors and L1B descendants; sub-lineage L1G is thought to have descended from L1B (16), so L1B-L1G mixing in the tree might be due to some misclassification of closely related sequences. This pattern was also apparent in the 3' ORFs fragment (ORF5 is embed in this larger fragment), though the clade containing the novel L1C-1-4-4 group became the closest sister to a clade containing the majority of L1A in the 3' ORFs tree. Intermixture of lineage groupings was more apparent in the three ORF1 fragments, suggesting some level of recombination between these genomic regions and ORF5. Although most taxa remained grouped by their ORF5 classification, numerous ancestral recombination were observed between lineage one sub-lineages. This observation was supported by a high number of transitions between traits (i.e., ORF5 lineage label), Bayes factors (Figure 2A), and ORF1ab tree topology (Figure 2B). The novel L1C-1-4-4 variant's evolutionary history was part of that phenomenon since it was a descendant of the major L1A clade in ORF1a-1 tree. An L1A virus collected in early 2018 (MN073102) was its closest related taxon in all ORF1 trees regardless of whether the L1C-1-4-4 clade was embedded in a larger L1C or L1A clade (Figure 2B), suggesting that this virus had a similar evolutionary and recombination history throughout this genomic region.

## Discussion

Exploratory analysis of the genome and evolutionary history of viruses causing atypical outbreaks is a key step to understanding their origin. Here, we analyze a set of whole genome sequences (WGSs) from an emerging PRRSV-2 variant and contextualize its evolution using publicly available WGSs from the U.S. swine industry. Our results suggest that the 2020–21 epidemic associated with the novel L1C-1-4-4 viruses arose from a recombinant ancestor of which most genomic parts derived from viruses whose ORF5 genes were classified as sub-lineage L1C. An ancestor of those viruses was estimated to have emerged around late 2018 to early 2019 with a slightly higher mutation rate than the average rate. Two samples from 2018, classified as the L1C-1-4-4 variant and L1A (MN073102) based on ORF5 gene, are the closest relatives of the 2020–21 epidemic variants, with phylogenetic placement varied according to which genomic was examined. The observed shift in phylogenetic clustering of the L1C-1-4-4 variant from L1C in the ORF5-based tree to L1A in ORF1A-based tree, combined with the inferred frequency of recombination estimated from the discrete trait analysis, highlights the role of recombination within L1 sub-lineages in shaping PRRSV-2 genetic diversity.

Interpretations from our analysis should consider some key limitations. First, samples from the NCBI database may not represent the diversity of the U.S. PRRSV-2 population since whole genome sequencing (WGS) is not a routine practice for disease surveillance because of its cost and availability. In addition, viruses with atypical clinical presentations in the field are more likely to undergo WGS. Thus, our recombination analysis only suggests the most likely parents or close relatives of the novel L1C-1-4-4 from amongst published sequences, which itself may be biased. In fact, the recombination detection was affected by data availability, as evidenced by several unknown parents of the novel L1C recombinant. Second, a fully recombination-free fragment, which is an ideal input for phylogenetic analysis, does not exist in the alignment because breakpoints are distributed across the genome. We alternatively used WGS-fragments with low frequencies of recombination to avoid recombination that may confound the genealogical tree. Genomic positions of such fragments nicely fit with three main protein coding regions of PRRSV-2 and other nidoviruses: ORF1a, ORF1b, and the nested set of multiple ORFs at the 3'-terminal (3' ORFs) (43). Last, the novel L1C-1-4-4 variant is defined by ORF5 genetic relatedness rather than clinical manifestation, and comparable data quantifying clinical aspects of disease were not available across data sources. Thus, an association between L1C-1-4-4's virulence and its evolution/recombination cannot be concluded from our study.

The inferred number of putative recombination events (trait transitions) from the discrete trait analysis reflect inter- and intra-lineage recombination between ORF5 and other genomic regions (i.e., ORF5 lineage was used as the discrete trait). From this analysis, we observe that recombination between lineages was rare, though this may be an artifact of the fact that the majority of included sequences belonged to a single lineage. However, recombination between sub-lineages within lineage one are more frequent, though still relatively uncommon. This corresponds to the mechanism of RNA recombination whereby the RNA polymerase is prone to switch from one RNA template to another that has a similar nucleotide sequence (7).

Additionally, recombination requires co-infection of the same cell, and viral prevalence will influence the likelihood that an animal is co-infected with two distinct viruses simultaneously. The prevalence of sub-lineages is temporally variable (12), which likely shapes opportunities for co-infection. Sub-lineages L1A, 1C, and 1H had the highest effective viral population sizes at the approximate tMRCA of the novel variant (12). Thus, the ancestor of the novel L1C-1-4-4 variant appears to have acquired each genomic portion from divergent viral subpopulations that were prevalent at the time. Recombination scanning along with phylogenetic tree analysis suggests that the majority of the 2020–2021 novel L1C-1-4-4 genomic fragments still derived from L1C viruses, while the proximal part of ORF1a, mostly nsp2, is genetically closer to viruses whose ORF5 is classified as L1A rather than L1C. This evidence coupled with the fact that nsp2 is the most variable gene in PRRSV-2 genome (44) explains why the novel L1C viruses clustered with viruses classified as L1A at the ORF5 level in the WGS tree in previous studies (10, 15).

The 2018 L1C-1-4-4 sample was included in this study because it carries an ORF5 gene closely related to the sequences associated with the 2020–2021 epidemic and was recovered from the same geographic area. However, some genomic parts as well as real world outbreak circumstances differ from the 2020–2021 epidemic. To our knowledge, there was no widespread PRRS outbreaks or heightened concern across the industry in connection with the 2018 virus, though anecdotally, field veterinarians noted that this particular virus transmitted readily between farms belonging to the same company and was challenging to control. The differential recombination profiles between the 2018 and 2020-21 L1C-1-4-4 viruses suggested by the RDP5 analysis were consistent with more robust phylogenetic analyses, which indicate that recombinant parents and phylogenetic position of the 2018 virus's ORF1b are different from the 2020–2021 sequences. Altogether, we hypothesize that both diverged in 2017 from the same recombinant ancestor that had a L1A-like ORF1a-1 fragment. The 2018 virus appears to be a result of an additional recombination event that appeared to leave very few progenies in our dataset. Other descendants kept evolving with or without recombination until they reached optimal fitness or a tipping point for exponential growth and became the 2020–2021 variant that is associated with the current outbreak.

An assessment of whether the acquisition of different WGS-fragments through recombination had a viral fitness benefit that allowed this variant to spread widely is beyond our limited understanding of the genetic determinants of pathogenicity and antigenicity. Therefore, we do not know the extent to which recombination contributed to the emergence or atypical clinical presentation of this virus. A study on SARS-CoV-2, a distant relative to PRRSV-2 in the same Nidovirales order, suggests the possibility that multi-strain recombination strengthens virulence (45). For PRRSV-2, all four genomic fragments we analyzed harbor at least one virulence-related gene. Mutations in nsp2, a part of both ORF1a-1 and ORF1a-2 fragments, are associated with target cell tropism of PRRSV-2 (46) and high fever in the host (47). RNA-dependent RNA polymerase (RdRp), a crucial component determining virus replication efficiency and pathogenicity (48), is encoded by nsp9 in ORF1b. Most of the 3'-terminal ORFs are transcribed and translated into the virus structural glycoprotein that directly interacts with either the target cell or host immune response (49, 50). Hypothetically, being able to rapidly shift antigenic phenotype through recombination may potentially confer a fitness advantage if it allows the virus to better evade population immunity. Genetic change in one of these genomic parts might be a key success of the novel L1C-1-4-4 variant but would need to be investigated by experimental studies such as targeted mutagenesis. However, our analysis better quantifies the contribution of recombination to PRRSV-2 genetic diversity and evolution, and points to the role of co-circulation of multiple variants within the same farm that may create conditions for recombination and selection for traits beneficial to the virus.

## Data Availability Statement

The datasets presented in this study can be found in online repositories. The names of the repository/repositories and accession number(s) can be found in the article/Supplementary Material.

## Author Contributions

NP and KV conceived and designed the study. NP performed data analysis and wrote the first draft of the manuscript. KV supervised the plan and findings of this work. CC and AR provided data for the analysis. NP, KV, MK, IP, and DM interpreted the results. All authors contributed to manuscript revision and approved the submitted version.

## Funding

Funding was provided by the joint NIFA-NSF-NIH Ecology and Evolution of Infectious Disease award 2019-67015-29918. This work was also supported by the University of Minnesota College of Veterinary Medicine Signature Programs, Grant Number MIN-62-133. NP was supported by the Royal Thai Government Scholarship. This project was partially funded by the University of Minnesota Swine Disease Eradication Center (SDEC) and the Swine Health Information Center (SHIC) as the funding agency for MSHMP.

## Conflict of Interest

The authors declare that the research was conducted in the absence of any commercial or financial relationships that could be construed as a potential conflict of interest.

## Publisher's Note

All claims expressed in this article are solely those of the authors and do not necessarily represent those of their affiliated organizations, or those of the publisher, the editors and the reviewers. Any product that may be evaluated in this article, or claim that may be made by its manufacturer, is not guaranteed or endorsed by the publisher.
