# Supplementary material for: Measuring How Recombination Re-shapes the Evolutionary History of PRRSV-2: A Genome-Based Phylodynamic Analysis of the Emergence of a Novel PRRSV-2 Variant
Source: Front Vet Sci. 2022 Mar 25;9:846904. doi: 10.3389/fvets.2022.846904 (PMC8990846; doi:10.3389/fvets.2022.846904)
Supplement: Supplementary file 1 [file Data_Sheet_1.docx]

**Supplementary Information**


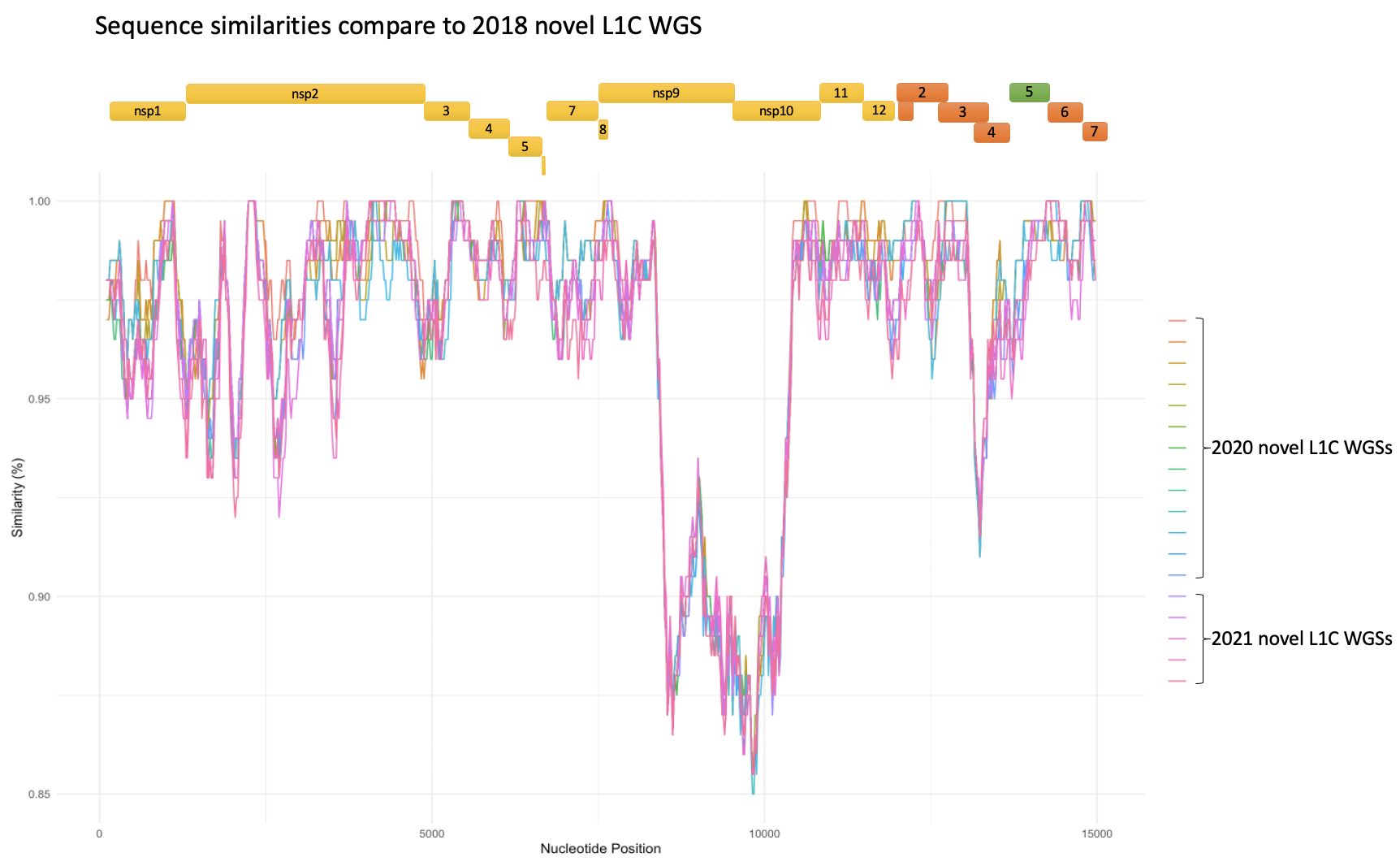


*Supplementary figure 1. Similarity plot between the 2018 (reference) and the 2020-21 (queries) L1C 1-4-4 WGSs*

*Supplementary table 1. List of the U.S. PRRSV-2 WGSs with collection year information used in this study*

| NCBI accession no. | ORF5-based lineage | State | Collection year | Sequences used for timed-tree and DTA |
| --- | --- | --- | --- | --- |
| OL963961 | novel L1C-1-4-4 | - | 2018 | 1 |
| OL963962 | novel L1C-1-4-4 | - | 2020 | 1 |
| OL963963 | novel L1C-1-4-4 | - | 2020 | 1 |
| OL963964 | novel L1C-1-4-4 | - | 2020 | 1 |
| OL963965 | novel L1C-1-4-4 | - | 2020 | 1 |
| OL963966 | novel L1C-1-4-4 | - | 2020 | 1 |
| OL963967 | novel L1C-1-4-4 | - | 2020 | 1 |
| OL963968 | novel L1C-1-4-4 | - | 2020 | 1 |
| OL963969 | novel L1C-1-4-4 | - | 2020 | 1 |
| OL963970 | novel L1C-1-4-4 | - | 2020 | 1 |
| OL963971 | novel L1C-1-4-4 | - | 2020 | 1 |
| OL963972 | novel L1C-1-4-4 | - | 2020 | 1 |
| OL963973 | novel L1C-1-4-4 | - | 2020 | 1 |
| OL963974 | novel L1C-1-4-4 | - | 2020 | 1 |
| OL963975 | novel L1C-1-4-4 | - | 2021 | 1 |
| OL963976 | novel L1C-1-4-4 | - | 2021 | 1 |
| OL963977 | novel L1C-1-4-4 | - | 2021 | 1 |
| OL963979 | novel L1C-1-4-4 | - | 2021 | 1 |
| OL963978 | novel L1C-1-4-4 | - | 2021 | 1 |
| HQ699067.1 | L9 | - | 2006 | 0 |
| JN654458.1 | L8 | - | 1996 | 0 |
| JN654459.1 | L1C | - | 2008 | 1 |
| JN660150.1 | L1B | - | 2008 | 0 |
| JQ087873.1 | L5 | - | 2010 | 1 |
| KC469618.1 | L5 | - | 1995 | 1 |
| KF632717.1 | L1C | - | 2012 | 1 |
| KF724397.1 | L1C | - | 2011 | 1 |
| KF724398.1 | L1C | - | 2011 | 1 |
| KF724399.1 | L9 | - | 2012 | 0 |
| KF724401.1 | L1C | - | 2012 | 1 |
| KF724402.1 | L1C | - | 2012 | 1 |
| KF724403.1 | L9 | - | 2012 | 0 |
| KF724404.1 | L8 | - | 2012 | 0 |
| KF724405.1 | L9 | - | 2012 | 0 |
| KF724406.1 | L9 | - | 2011 | 0 |
| KF724407.1 | L6 | - | 2013 | 0 |
| KF724409.1 | L1C | - | 2013 | 1 |
| KF724410.1 | L1C | - | 2013 | 1 |
| KF724411.1 | L1C | - | 2013 | 1 |
| KF724412.1 | L1G | - | 2013 | 1 |
| KF724413.1 | L5 | - | 2013 | 1 |
| KP283399.1 | L1G | - | 2012 | 1 |
| KP283400.1 | L1G | - | 2012 | 1 |
| KP283401.1 | L1C | - | 2012 | 1 |
| KP283402.1 | L1C | - | 2013 | 1 |
| KP283403.1 | L1C | - | 2012 | 1 |
| KP283404.1 | L1C | - | 2011 | 1 |
| KP283405.1 | L1G | - | 2012 | 1 |
| KP283406.1 | L1G | - | 2012 | 1 |
| KP283407.1 | L1G | - | 2012 | 1 |
| KP283408.1 | L1G | - | 2012 | 1 |
| KP283409.1 | L1C | - | 2012 | 1 |
| KP283410.1 | L1C | - | 2012 | 1 |
| KP283411.1 | L1C | - | 2012 | 1 |
| KP283412.1 | L1C | - | 2012 | 1 |
| KP283413.1 | L1C | - | 2012 | 1 |
| KP283414.1 | L1A | - | 2012 | 1 |
| KP283415.1 | L1C | - | 2012 | 1 |
| KP283416.1 | L1C | - | 2012 | 0 |
| KR534893.1 | L1C | OH | 2013 | 0 |
| KR534894.1 | L1A | OH | 2015 | 1 |
| KT207837.1 | L9 | - | 2012 | 0 |
| KT257944.1 | L5 | IA | 2014 | 0 |
| KT257945.1 | L5 | IA | 2014 | 0 |
| KT257946.1 | L5 | IA | 2014 | 0 |
| KT257947.1 | L5 | IA | 2014 | 0 |
| KT257948.1 | L8 | MN | 2014 | 0 |
| KT257949.1 | L8 | MN | 2014 | 0 |
| KT257950.1 | L8 | MN | 2014 | 0 |
| KT257951.1 | L8 | MN | 2014 | 0 |
| KT257952.1 | L8 | MN | 2014 | 0 |
| KT257953.1 | L8 | MN | 2014 | 0 |
| KT257954.1 | L1E | KS | 2014 | 1 |
| KT257955.1 | L1E | CO | 2014 | 1 |
| KT257956.1 | L1H | KS | 2014 | 0 |
| KT257957.1 | L6 | KS | 2014 | 0 |
| KT257958.1 | L1E | KS | 2014 | 1 |
| KT257959.1 | L1B | - | 2014 | 1 |
| KT257960.1 | L1B | - | 2014 | 1 |
| KT257961.1 | L1B | - | 2014 | 1 |
| KT257962.1 | L1B | - | 2014 | 1 |
| KT257963.1 | L1B | - | 2014 | 1 |
| KT257964.1 | L1B | - | 2014 | 1 |
| KT257965.1 | L1B | - | 2014 | 1 |
| KT257966.1 | L1A | NC | 2014 | 1 |
| KT257967.1 | L1A | IA | 2014 | 1 |
| KT257968.1 | L1A | IA | 2014 | 1 |
| KT257969.2 | L1C | - | 2014 | 1 |
| KT257970.1 | L1C | - | 2014 | 1 |
| KT257971.1 | L1C | - | 2014 | 1 |
| KT257972.1 | L1C | - | 2014 | 1 |
| KT257973.1 | L1C | - | 2014 | 1 |
| KT257974.1 | L1A | - | 2014 | 1 |
| KT257975.1 | L1G | - | 2014 | 1 |
| KT257976.1 | L1A | - | 2014 | 1 |
| KT257977.1 | L1C | IA | 2014 | 1 |
| KT257978.1 | L1D | - | 2014 | 0 |
| KT257979.1 | L1C | - | 2014 | 0 |
| KT257980.1 | L1C | - | 2014 | 0 |
| KT257981.1 | L1C | - | 2014 | 0 |
| KT257982.1 | L1C | - | 2014 | 0 |
| KT257983.1 | L1C | IA | 2014 | 1 |
| KT257984.1 | L1C | - | 2014 | 1 |
| KT257985.1 | L1A | - | 2014 | 1 |
| KT257986.1 | L1C | - | 2014 | 1 |
| KT257987.1 | L1G | IA | 2014 | 1 |
| KT257988.1 | L1A | - | 2014 | 1 |
| KT257989.1 | L1A | - | 2014 | 1 |
| KT257990.1 | L1A | - | 2014 | 1 |
| KT257991.1 | L1A | - | 2014 | 1 |
| KT257992.1 | L1G | MO | 2014 | 1 |
| KT257993.1 | L1E | IA | 2014 | 0 |
| KT257994.1 | L1D | NE | 2014 | 0 |
| KT257995.1 | L1D | NE | 2014 | 0 |
| KT257996.1 | L1D | NE | 2014 | 0 |
| KT257997.1 | L1D | NE | 2014 | 0 |
| KT257998.1 | L1D | NE | 2014 | 0 |
| KT257999.1 | L1H | CO | 2014 | 0 |
| KT258000.1 | L1H | CO | 2014 | 0 |
| KT258001.1 | L1E | IA | 2014 | 0 |
| KT258002.1 | L1E | IA | 2014 | 0 |
| KT258003.1 | L1E | IA | 2014 | 0 |
| KT258004.1 | L1E | IA | 2014 | 0 |
| KT258005.1 | L1A | NE | 2014 | 1 |
| KT258006.1 | L1G | NC | 2014 | 1 |
| KT258007.1 | L1G | NE | 2014 | 1 |
| KT258008.1 | L1G | MN | 2014 | 1 |
| KT258009.1 | L1H | IA | 2014 | 0 |
| KT581982.1 | L1C | - | 2014 | 1 |
| KU131557.1 | L1D | - | 2011 | 0 |
| KU131559.1 | L5 | - | 2004 | 0 |
| KU131561.1 | L5 | - | 2002 | 0 |
| KU131563.1 | L8 | - | 1998 | 0 |
| KU131564.1 | L5 | - | 1995 | 0 |
| KU131565.1 | L9 | - | 1995 | 0 |
| KU131566.1 | L1D | - | 2011 | 0 |
| KU131568.1 | L1D (Prevacent vaccine) | - | 2011 | 0 |
| KU318406.1 | L5 | - | 2015 | 1 |
| KX192112.1 | L1A | NE | 2016 | 1 |
| KX192113.1 | L1A | NE | 2016 | 1 |
| KX192114.1 | L1A | NE | 2016 | 1 |
| KX192115.1 | L1A | NE | 2016 | 1 |
| KX192116.1 | L1A | NE | 2016 | 1 |
| KX192117.1 | L1A | NE | 2016 | 1 |
| KX192118.1 | L1A | NE | 2016 | 1 |
| KX192119.1 | L1A | NE | 2016 | 1 |
| KX462792.1 | L5 | - | 2012 | 1 |
| KY348847.1 | L9 | - | 1998 | 0 |
| KY348848.1 | L1F | - | 2004 | 0 |
| KY348849.1 | L1D | - | 2001 | 0 |
| KY348850.1 | L9 | - | 2000 | 0 |
| KY348851.1 | L5 | - | 2000 | 0 |
| KY348852.1 | L8 | - | 2000 | 1 |
| KY348853.1 | L5 | - | 2000 | 0 |
| MF326985.1 | L1A | IA | 2014 | 1 |
| MF326986.1 | L1A | IA | 2015 | 1 |
| MF326987.1 | L1A | IA | 2015 | 1 |
| MF326988.1 | L1A | IA | 2013 | 1 |
| MF326989.1 | L1A | IA | 2014 | 1 |
| MF326990.1 | L1A | NC | 2014 | 1 |
| MF326991.1 | L1A | NC | 2014 | 1 |
| MF326992.1 | L1A | IN | 2014 | 1 |
| MF326993.1 | L1A | OH | 2014 | 1 |
| MF326994.1 | L1A | OH | 2014 | 1 |
| MF326995.1 | L1A | IA | 2014 | 1 |
| MF326996.1 | L1A | IA | 2015 | 1 |
| MF326997.1 | L1A | IA | 2015 | 1 |
| MF326998.1 | L1A | NC | 2015 | 1 |
| MF326999.1 | L1A | NC | 2015 | 1 |
| MF327000.1 | L1A | IA | 2015 | 1 |
| MF327001.1 | L1A | IA | 2015 | 1 |
| MF526964.1 | L1A | IN | 2017 | 1 |
| MF526965.1 | L1C | IN | 2017 | 1 |
| MK796164.1 | L1C | - | 2018 | 0 |
| MK796165.1 | L8 | - | 2018 | 0 |
| MK837936.1 | L8 | - | 1996 | 0 |
| MK860181.1 | L1A | NC | 2015 | 1 |
| MN073081.1 | L1A | - | 2018 | 1 |
| MN073082.1 | L1A | - | 2018 | 1 |
| MN073083.1 | L1A | - | 2018 | 1 |
| MN073084.1 | L1A | - | 2018 | 1 |
| MN073086.1 | L1A | - | 2017 | 1 |
| MN073088.1 | L1A | - | 2017 | 1 |
| MN073089.1 | L1A | - | 2017 | 1 |
| MN073092.1 | L1A | - | 2017 | 1 |
| MN073095.1 | L1A | - | 2017 | 1 |
| MN073097.1 | L1A | - | 2017 | 1 |
| MN073098.1 | L1A | - | 2017 | 1 |
| MN073099.1 | L1A | - | 2017 | 1 |
| MN073100.1 | L1A | - | 2017 | 1 |
| MN073102.1 | L1A | - | 2018 | 1 |
| MN073111.1 | L1C | - | 2017 | 1 |
| MN073112.1 | L1C | - | 2017 | 1 |
| MN073113.1 | L1C | - | 2017 | 1 |
| MN073114.1 | L1C | - | 2016 | 1 |
| MN073115.1 | L1C | - | 2016 | 1 |
| MN073116.1 | L1C | - | 2017 | 1 |
| MN073117.1 | L1C | - | 2017 | 1 |
| MN073118.1 | L1C | - | 2017 | 1 |
| MN073119.1 | L1C | - | 2017 | 0 |
| MN073120.1 | L1C | - | 2017 | 0 |
| MN073121.1 | L1C | - | 2017 | 0 |
| MN073122.1 | L1C | - | 2018 | 0 |
| MN073123.1 | L1B | - | 2017 | 1 |
| MN073124.1 | L1B | - | 2017 | 1 |
| MN073127.1 | L1F | - | 2018 | 0 |
| MN073129.1 | L7 | - | 2018 | 0 |
| MN073130.1 | L5 | - | 2018 | 1 |
| MN073131.1 | L5 | - | 2018 | 1 |
| MN073132.1 | L5 | - | 2018 | 1 |
| MN073133.1 | L5 | - | 2016 | 1 |
| MN073136.1 | L5 | - | 2017 | 1 |
| MN073137.1 | L5 | - | 2017 | 1 |
| MN073138.1 | L5 | - | 2016 | 1 |
| MN073139.1 | L5 | - | 2016 | 1 |
| MN073140.1 | L5 | - | 2017 | 1 |
| MN073141.1 | L5 | - | 2017 | 0 |
| MN073142.1 | L5 | - | 2017 | 0 |
| MN073143.1 | L5 | - | 2017 | 0 |
| MN073144.1 | L5 | - | 2017 | 0 |
| MN073145.1 | L5 | - | 2017 | 0 |
| MN073146.1 | L5 | - | 2017 | 0 |
| MN073147.1 | L5 | - | 2017 | 1 |
| MN073148.1 | L5 | - | 2017 | 0 |
| MN073149.1 | L5 | - | 2017 | 0 |
| MN073150.1 | L5 | - | 2017 | 1 |
| MN073151.1 | L5 | - | 2017 | 1 |
| MN073155.1 | L8 | - | 2016 | 0 |
| MN073157.1 | L8 | - | 2018 | 0 |
| MN073158.1 | L8 | - | 2017 | 1 |
| MN073159.1 | L8 | - | 2017 | 1 |
| MN073160.1 | L8 | - | 2017 | 1 |
| MN073161.1 | L8 | - | 2017 | 0 |
| MN073162.1 | L8 | - | 2017 | 0 |
| MN073164.1 | L8 | - | 2017 | 0 |
| MN073165.1 | L8 | - | 2017 | 0 |
| MN073168.1 | L8 | - | 2016 | 0 |
| MN073169.1 | L8 | - | 2017 | 0 |
| MN073170.1 | L8 | - | 2017 | 0 |
| MN073171.1 | L8 | - | 2017 | 0 |
| MN073172.1 | L1D | - | 2018 | 0 |
| MN073173.1 | L1E | - | 2018 | 0 |
| MN073177.1 | L1H | - | 2017 | 0 |
| MN073178.1 | L1H | - | 2017 | 0 |
| MN073180.1 | L1H | - | 2018 | 0 |
| MN175677.1 | L1A | - | 2016 | 1 |
| MT269876.1 | L5 | - | 2009 | 0 |
| MT269877.1 | L1F | - | 2006 | 0 |
| MT269878.1 | L1C | - | 2016 | 1 |
| MT269879.1 | L1A | - | 2016 | 1 |
| MT376723.1 | L1A | OH | 2016 | 1 |


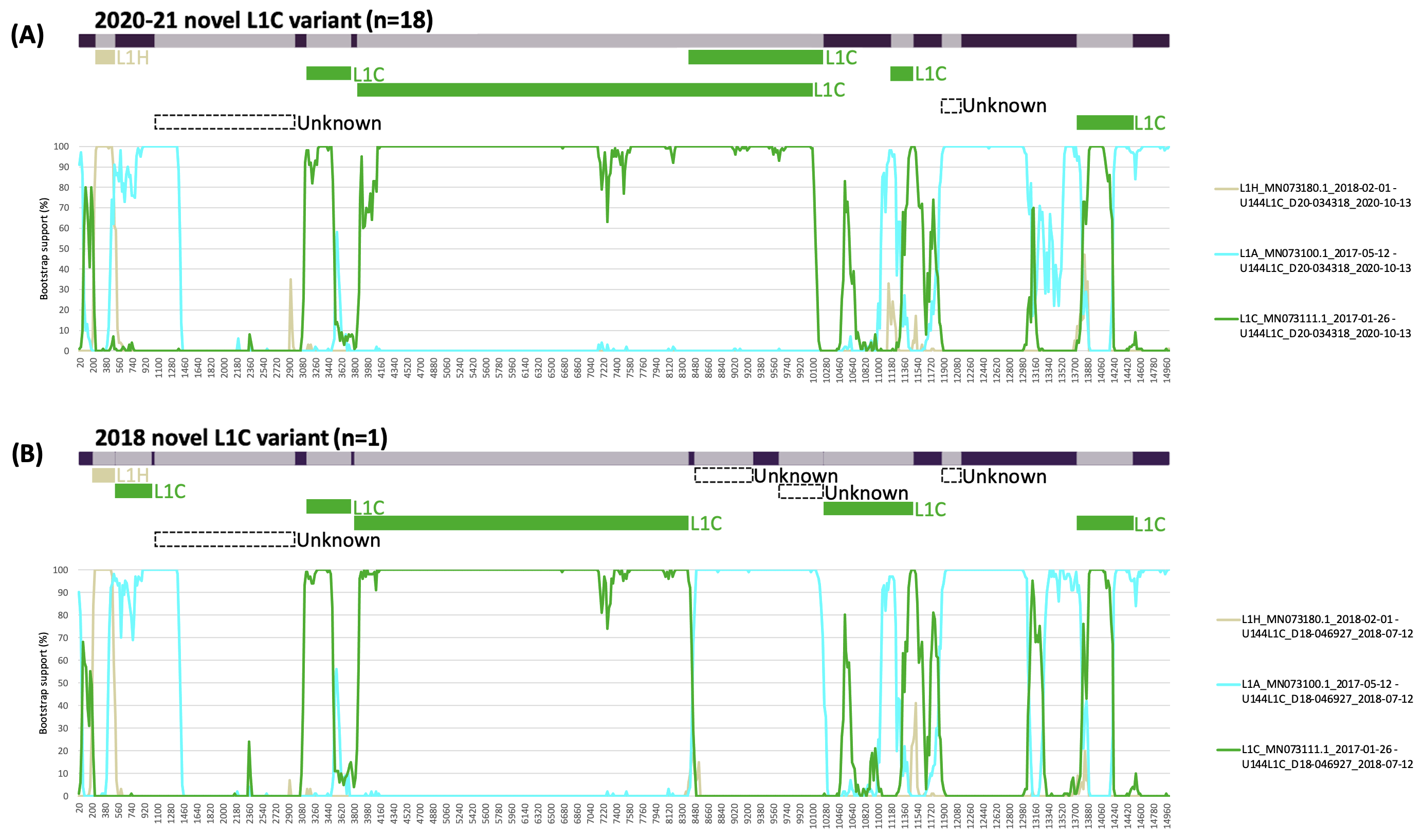


*Supplementary figure 2. BOOTSCAN plots showing putative recombination parents of the 2020-21 (A) and the 2018 L1C 1-4-4 WGSs (B) across genomic position compared to the full recombination plots summarized from the 7 detection methods. The bootstrap value (%) supporting the clustering of the novel L1C 1-4-4 recombinants – each parent was shown on the y-axis. Each putative parent was colored based on ORF5 gene-based classification; L1C (green), L1A (blue), and L1H (beige).*

| **position in alignment**  *Supplementary table 2. Putative recombination profile of the novel L1C 1-4-4 WGSs detected by the 7 methods in RDP5* | **gene** | **number of the novel L1C recombinants** | **Minor Parental Sequence(s)** | **Major Parental Sequence(s)** | **Detection Methods** | | | | | | |
| --- | --- | --- | --- | --- | --- | --- | --- | --- | --- | --- | --- |
|  |  |  |  |  | RDP | GENECONV | Bootscan | Maxchi | Chimaera | SiSscan | 3Seq |
| 188-506 | nsp1 | 19/19(all novel L1C) | L1H_MN073180.1 | L1A_MN073102.1 | 2.3632 𝗑 10^-19^ | 2.744 𝗑 10^-13^ | 1.2804 𝗑 10^-19^ | 4.6843 𝗑 10^-6^ | 1.6803 𝗑 10^-6^ | 3.554 𝗑 10^-5^ | 3.2907 𝗑 10^-13^ |
| 507-1022 | nsp1 | 1/19(only 2018 sequence) | L1C_MF526965.1 | Unknown*(L1A_KT257966.1,L1A_MF326990.1,L1A_MF326998.1,L1A_MN073084.1,L1A_MN073086.1,L1A_MN073088.1,L1A_MN073095.1) | 0.00039217 | NS** | 0.00164049 | 1.1918 𝗑 10^-5^ | 0.01518259 | NS | 5.6588 𝗑 10^-7^ |
| 1038-3010 | nsp1-2 | 19/19(all novel L1C) | Unknown(L1C_KP283416.1,L1A_MF326993.1,L1C_KR534893.1,L1C_KT257979.1,L1C_KT257980.1,L1C_KT257981.1,L1C_KT257982.1,L1C_KT581982.1,L1C_MN073119.1,L1C_MN073120.1,L1C_MN073121.1) | L1A_KP283414.1,L1C_JN654459.1,L1C_KF724409.1,L1C_KF724410.1,L1C_KP283401.1,L1C_KP283402.1,L1C_KP283403.1,L1C_KP283404.1,L1C_KP283410.1,L1C_MN073116.1,L1C_MN073117.1,L1C_MN073118.1 | 1.1591 𝗑 10^-85^ | 3.3651 𝗑 10^-87^ | 3.388 𝗑 10^-54^ | 2.6093 𝗑 10^-31^ | 1.7083 𝗑 10^-31^ | 5.8983 𝗑 10^-55^ | 1.4449 𝗑 10^-38^ |
| 3135-3780 | nsp2 | 19/19(all novel L1C) | L1C_KT581982.1 | L1A_KT257988.1,L1A_KT257989.1,L1A_KT257990.1,L1A_KT257991.1 | 8.7186 𝗑 10^-28^ | 3.3644 𝗑 10^-31^ | 1.6693 𝗑 10^-41^ | 1.961 𝗑 10^-16^ | 3.5081 𝗑 10^-18^ | 2.7754 𝗑 10^-19^ | 3.1557 𝗑 10^-38^ |
| 3802-8442 | nsp2-9 | 19/19(all novel L1C) | L1C_MN073111.1,L1C_MN073112.1 | L1A_MN073100.1 | 6.667 𝗑 10^-148^ | 1.821 𝗑 10^-148^ | 1.152 𝗑 10^-126^ | 2.0972 𝗑 10^-43^ | 3.2853 𝗑 10^-44^ | 3.0092 𝗑 10^-67^ | 2.958 𝗑 10^-112^ |
| 8443-11554 | nsp9-12 | 18/19(all except 2018 sequences) | L1C_MN073114.1,L1C_MN073115.1 | L1A_MN073097.1 | 6.6777 𝗑 10^-13^ | 1.2441 𝗑 10^-8^ | 1.2762 𝗑 10^-12^ | 6.0304 𝗑 10^-7^ | 4.3242 𝗑 10^-5^ | 1.7739 𝗑 10^-6^ | 5.2322 𝗑 10^-10^ |
| 8452-11230 | nsp9-11 | 18/19(all except 2018 sequences) | L1C_MN073114.1,L1C_MN073115.1 | U144L1C_D18-046927 | 2.083 𝗑 10^-108^ | 2.5278 𝗑 10^-86^ | 2.807 𝗑 10^-103^ | 3.0471 𝗑 10^-28^ | 3.0869 𝗑 10^-28^ | 5.2367 𝗑 10^-32^ | 2.724 𝗑 10^-91^ |
| 8522-9358 | nsp9 | 1/19(only 2018 sequence) | Unknown(L1G_KP283400.1,L1G_KP283399.1,L1G_KP283408.1,L1G_KT258008.1) | L1A_MF326994.1,L1A_KR534894.1,L1A_KT257966.1,L1A_KT257967.1,L1A_KT257968.1,L1A_KT257976.1,L1A_KT258005.1,L1A_KX192112.1,L1A_KX192113.1,L1A_KX192114.1,L1A_KX192115.1,L1A_KX192116.1,L1A_KX192117.1,L1A_KX192118.1,L1A_KX192119.1,L1A_MF326985.1,L1A_MF326986.1,L1A_MF326987.1,L1A_MF326990.1,L1A_MF326991.1,L1A_MF326995.1,L1A_MF326996.1,L1A_MF326997.1,L1A_MF326998.1,L1A_MF326999.1,L1A_MF327000.1,L1A_MF327001.1,L1A_MK860181.1,L1A_MN073084.1,L1A_MN073086.1,L1A_MN073088.1,L1A_MN073089.1,L1A_MN073092.1,L1A_MN073095.1,L1A_MN073098.1,L1A_MN073099.1,L1A_MN175677.1,L1A_MT269879.1,L1A_MT376723.1,L1C_MF526965.1 | 2.6157 𝗑 10^-11^ | NS | 2.2984 𝗑 10^-11^ | 3.0108 𝗑 10^-5^ | 1.0899 𝗑 10^-6^ | 4.4837 𝗑 10^-5^ | 1.6454 𝗑 10^-13^ |
| 9684-10313 | nsp10 | 1/19(only 2018 sequence) | Unknown(L1F_MT269877.1,L1F_KY348848.1) | L1C_KF632717.1 | 5.5983 𝗑 10^-9^ | 2.7539 𝗑 10^-5^ | 2.0963 𝗑 10^-11^ | 2.7359 𝗑 10^-6^ | 4.3888 𝗑 10^-7^ | 1.2764 𝗑 10^-10^ | 1.4716 𝗑 10^-7^ |
| 10314-11554 | nsp10-12 | 1/19(only 2018 sequence) | L1C_MN073114.1,L1C_MN073115.1 | L1A_KT257990.1,L1A_KT257988.1,L1A_KT257989.1,L1A_KT257991.1 | 2.1769 𝗑 10^-29^ | 1.4218 𝗑 10^-19^ | 1.1251 𝗑 10^-29^ | 6.5286 𝗑 10^-16^ | 2.5242 𝗑 10^-9^ | 2.0791 𝗑 10^-17^ | 6.493 𝗑 10^-21^ |
| 11968-12217 | ORF2 | 19/19(all novel L1C) | Unknown(L8_MN073165.1,L5_KU131561.1,L5_KY348853.1,L6_KF724407.1,L8_JN654458.1,L8_KU131563.1,L8_KY348852.1,L8_MK796165.1,L8_MK837936.1,L8_MN073157.1,L8_MN073158.1,L8_MN073159.1,L8_MN073160.1,L8_MN073161.1,L8_MN073162.1,L8_MN073164.1,L8_MN073168.1,L8_MN073169.1,L8_MN073170.1,L8_MN073171.1,L9_KT207837.1,L9_KU131565.1,L9_KY348847.1) | L1C_KP283401.1,L1C_KF632717.1,L1C_KF724409.1,L1C_KF724410.1,L1C_KF724411.1,L1C_KP283402.1,L1C_KP283403.1,L1C_KP283404.1,L1C_KP283409.1,L1C_KP283410.1,L1C_KP283413.1,L1C_MN073121.1,L1C_MT269878.1 | 1.3832 𝗑 10^-5^ | NS | 1.8005 𝗑 10^-5^ | 0.00030582 | 0.00011315 | 2.5529 𝗑 10^-5^ | 8.3087 𝗑 10^-9^ |
| 13802-14582 | ORF5-6 | 19/19(all novel L1C) | L1C_KF724410.1,L1C_KF724409.1,L1C_KF724411.1,L1C_KP283401.1,L1C_KP283409.1,L1C_KP283410.1,L1C_MN073116.1,L1C_MN073117.1,L1C_MN073118.1 | L1A_MF326985.1 | 2.0973 𝗑 10^-27^ | 1.7438 𝗑 10^-22^ | 6.3121 𝗑 10^-27^ | 6.2024 𝗑 10^-12^ | 1.5666 𝗑 10^-13^ | 5.2422 𝗑 10^-14^ | 8.433 𝗑 10^-25^ |

**Unknown = The sequence listed as unknown was used to infer the existence of a missing parental sequence. (Only one parent and a recombinant need be in the alignment for a recombination event to be detectable.)*

***NS = No significant P-value was recorded for this recombination event using the particular method.*

*Supplementary table 3. Temporal signal of PRRSV-2 WGS fragment trees*

| WGS  fragment | Best-fitting root method | | | | | | | |
| --- | --- | --- | --- | --- | --- | --- | --- | --- |
|  | Heuristic residual mean squared | | Residual mean squared | | Correlation | | R-squared | |
|  | r* | R^2^ | r | R^2^ | r | R^2^ | r | R^2^ |
| ORF1a-1 | 0.3817 | 0.1457 | 0.3817 | 0.1457 | 0.3875 | 0.1502 | 0.3875 | 0.1502 |
| ORF1a-2 | -0.0256 | 6.56 𝗑 10^-4^ | -0.0256 | 6.56 𝗑 10^-4^ | 0.2677 | 7.17 𝗑 10^-2^ | 0.2677 | 7.17 𝗑 10^-2^ |
| ORF1b | 0.3438 | 0.1182 | 0.3722 | 0.1385 | 0.4447 | 0.1978 | 0.4447 | 0.1978 |
| 3'ORFs | 0.06546 | 4.29 𝗑 10^-3^ | 0.06546 | 4.29 𝗑 10^-3^ | 0.3411 | 0.1164 | -0.3905 | 0.1525 |

**r = Pearson’s correlation coefficient between root-to-tip divergence and time*
